# Crosstalk between Macrophages and Myxoid Liposarcoma Cells Increases Spreading and Invasiveness of Tumor Cells

**DOI:** 10.3390/cancers13133298

**Published:** 2021-06-30

**Authors:** Michele Minopoli, Sabrina Sarno, Lucia Cannella, Salvatore Tafuto, Gosuè Scognamiglio, Michele Gallo, Flavio Fazioli, Rosa Azzaro, Gaetano Apice, Biagio De Angelis, Elena Tamborini, Cecilia Garofalo, Ymera Pignochino, Laura Mercatali, Toni Ibrahim, Rita Falcioni, Beatrice Valenti, Roberta Maestro, Katia Scotlandi, Annarosaria De Chiara, Maria Vincenza Carriero

**Affiliations:** 1 Neoplastic Progression Unit, Istituto Nazionale Tumori IRCCS ‘Fondazione G. Pascale’, 80131 Naples, Italy; m.minopoli@istitutotumori.na.it (M.M.); sabrina.sarno@istitutotumori.na.it (S.S.); 2 Medical Oncology Unit, Istituto Nazionale Tumori IRCCS ‘Fondazione G. Pascale’, 80131 Naples, Italy; l.cannella@istitutotumori.na.it (L.C.); s.tafuto@istitutotumori.na.it (S.T.); g.apice@istitutotumori.na.it (G.A.); 3 Pathology Unit, Istituto Nazionale Tumori IRCCS ‘Fondazione G. Pascale’, 80131 Naples, Italy; giosue.scognamiglio@istitutotumori.na.it (G.S.); a.dechiara@istitutotumori.na.it (A.D.C.); 4 Musculoskeletal Surgery Unit, Istituto Nazionale Tumori IRCCS ‘Fondazione G. Pascale’, 80131 Naples, Italy; m.gallo@istitutotumori.na.it (M.G.); f.fazioli@istitutotumori.na.it (F.F.); 5 Transfusion Medicine Unit, Istituto Nazionale Tumori IRCCS ‘Fondazione G. Pascale’, 80131 Naples, Italy; r.azzaro@istitutotumori.na.it; 6 Department of Onco-Haematology and Cell and Gene Therapy Unit, Bambino Gesù Children’s Hospital, IRCCS, 00140 Rome, Italy; biagio.deangelis@opbg.net; 7 Department of Pathology, Fondazione IRCCS Istituto Nazionale dei Tumori, 20133 Milan, Italy; Elena.Tamborini@istitutotumori.mi.it; 8 Advanced Translational Research Laboratory, Veneto Institute of Oncology IOV—IRCCS, 35128 Padua, Italy; cecilia.garofalo@iov.veneto.it; 9 Department of Clinical and Biological Sciences, University of Turin, 10124 Torino, Italy; ymera.pignochino@ircc.it; 10 Division of Medical Oncology, Candiolo Cancer Institute, FPO-IRCCS, 10060 Candiolo, Italy; 11 Osteoncology and Rare Tumors Center, Istituto Romagnolo per lo Studio dei Tumori “Dino Amadori” (IRST) IRCCS, 47010 Meldola, Italy; laura.mercatali@irst.emr.it (L.M.); toni.ibrahim@irst.emr.it (T.I.); 12 Department of Research, Diagnosis and Innovative Technology, IRCCS Regina Elena National Cancer Institute, 00128 Rome, Italy; rita.falcioni@ifo.gov.it; 13 Oncogenetics and Functional Oncogenomics, Centro di Riferimento Oncologico di Aviano (CRO Aviano) IRCCS, National Cancer Institute, 33081 Aviano, Italy; beatrice.valenti@cro.it (B.V.); rmaestro@cro.it (R.M.); 14 Laboratory of Experimental Oncology, IRCCS Istituto Ortopedico Rizzoli, 40136 Bologna, Italy; katia.scotlandi@ior.it

**Keywords:** myxoid liposarcoma, tumor microenvironment, tumor associated macrophages, tumor-infiltrating lymphocytes

## Abstract

**Simple Summary:**

Myxoid liposarcoma (MLPS) is the second most common subtype of liposarcoma, occurs predominantly in the extremities, and tends either to recur locally and metastasize to unusual soft tissue locations. To date, the mechanisms of invasion and metastasis of MLPS remain unclear, and yet, there is a high need to identify new prognostic biomarkers to enable the development of novel targeted therapeutic strategies. This study firstly aimed to assess the role of immune cellular components that infiltrate MLPS tissues. Our data show that high grade, heavily vascularized MLPS tissues exhibit T lymphocyte-poor and M2-like macrophage-rich phenotypes, while low grade MLPS tissues are mainly infiltrated by T lymphocytes. In line with these findings, evidence is shown that a crosstalk occurring between MLPS cells and macrophages exists as MLPS cells drive an M2-like phenotype in monocytes which in turn, increase the invasive capability of MLPS cells.

**Abstract:**

Myxoid liposarcoma (MLPS) is the second most common subtype of liposarcoma and has tendency to metastasize to soft tissues. To date, the mechanisms of invasion and metastasis of MLPS remain unclear, and new therapeutic strategies that improve patients’ outcomes are expected. In this study, we analyzed by immunohistochemistry the immune cellular components and microvessel density in tumor tissues from patients affected by MLPS. In order to evaluate the effects of primary human MLPS cells on macrophage polarization and, in turn, the ability of macrophages to influence invasiveness of MLPS cells, non-contact and 3D organotypic co-cultures were set up. High grade MLPS tissues were found heavily vascularized, exhibited a CD3, CD4, and CD8 positive T lymphocyte-poor phenotype and were massively infiltrated by CD163 positive M2-like macrophages. Conversely, low grade MLPS tissues were infiltrated by a discrete amount of CD3, CD4, and CD8 positive T lymphocytes and a scarce amount of CD163 positive macrophages. Kaplan–Meier analysis revealed a shorter Progression Free Survival in MLPS patients whose tumor tissues were highly vascularized and heavily infiltrated by CD163 positive macrophages, indicating a clear-cut link between M2-like macrophage abundance and poor prognosis in patients. Moreover, we documented that, in co-culture, soluble factors produced by primary human MLPS cells induce macrophage polarization toward an M2-like phenotype which, in turn, increases MLPS cell capability to spread into extracellular matrix and to cross endothelial monolayers. The identification of M2-like polarization factors secreted by MLPS cells may allow to develop novel targeted therapies counteracting MLPS progression.

## 1. Introduction

Myxoid Liposarcoma (MLPS) is the second most common type of liposarcoma, representing 20–30% of all liposarcomas and an about 5% of all adult soft tissue sarcomas. These tumors occur not only in adults but also in a younger population, with a peak of incidence in the fourth and fifth decade of life and equal distribution between men and women [1,2]. Histologically, MLPS is characterized by a mixture of round to oval non-lipogenic cells and small ring lipoblasts dispersed in an abundant myxoid matrix stroma with a thin, delicate, so called “chicken wire” capillary network [3]. Different factors have been evaluated in an effort to predict the prognosis at initial diagnosis of MLPS, including patient age, tumor size and depth, vascularization, grading, necrosis, mitotic rate, and p53 overexpression [4,5,6]. Nowadays, the hyper-cellularity is considered the most important one affecting the development of distant metastases, and a greater amount of round cells well correlate with a higher histologic grade and poorer prognosis. A cellular overlap > 5%, decreased myxoid matrix, increased nuclear grade, and high mitotic activity hallmark high-grade MLPS [2]. The recurrent (12;16) (q13;p11) chromosome translocation that results in the *FUS-DDIT3* gene fusion has a 95% incidence, while the variant (12;22)(q13;q12), in which *DDIT3* rearranges with *EWSR1,* occurs in only 5% of MLPS cases [7,8]. More than 50% of cases carry *TERT* promoter mutations [9]. Other less frequent genetic aberrations described in MLPS include *PI3KCA* mutations [10,11], homozygous loss of PTEN, high expression of RET, IGF1R and IGF2 [12,13]. The metastatic behavior of MLPS is characterized by a propensity of tumor cells to spread to extra-pulmonary locations with a predilection to the bone, particularly spine and abdominal cavity [14,15]. Metastases occur in 30–60% of MLPS cases, and the prognosis of these patients remains poor [16]. Actually, wide surgical resection, combined with or without radiotherapy, is the treatment of choice for localized disease, whereas several clinical trials with molecular targeted agents are currently under investigation for patients with advanced or metastatic disease [17,18,19]. Based on these considerations, the identification of new biomarker of tumor progression as well as new therapeutic strategies are an unmet need, especially for patients with advanced disease. 

Some evidence indicates that trabectedin may be a therapeutic option for MLPS patients. The mechanism of action of this drug is complex, and it seems to rely not only on DNA damage but also on modulation of tumor microenvironment, including infiltrating macrophages and intra-tumor vascularization [20]. 

In the last ten years, the emerging role of tumor microenvironment (TME) in cancer progression induced researchers to consider solid tumors as complex ecosystems, in which the TME immune cells may both counteract or promote tumor progression, depending on their nature and their functional state [21,22]. It has been shown in several solid tumors that cytokines and chemokines secreted by cancer cells may recruit circulating leukocytes from blood into the neoplastic tissues, and initiate a complex cross-talk with tumor cells, exerting cytotoxic or, alternatively, pro-tumor activity [23,24,25]. In this regard, several reports highlight that immune cells infiltrating solid tumors impact on clinical outcomes of patients. High levels of CD8+ cytotoxic T lymphocytes and CD4+ helper T cells are in general favorable prognostic indicators whereas other immune cells, such as regulatory T cells and tumor-associated macrophages (TAM)s, may promote tumor progression [26]. More recently, molecular profiling studies allowed to identify a number of immune therapeutic targets in bone sarcomas [27]. Otherwise, most of soft tissue sarcomas are considered non-immunogenic [1], few reports investigating the composition of TME in soft tissue sarcomas have been published, and clinical responses in trials with checkpoint inhibitors still remain unsatisfactory [28,29,30,31,32]. 

The main focus of this study was to quantify and characterize the cellular composition of the tumor immune infiltrate in a large cohort of MLPS cases and to explore the association of cell subtype with the histologic grade, microvessel density, and the Progression Free Survival (PFS). Moreover, the contribution of primary human MLPS cells in affecting macrophages polarization toward an M2-like phenotype and, in turn, the macrophage ability to modulate spreading and invasiveness of MLPS cells have been investigated by non-contact and organotypic co-cultures. 

## 2. Materials and Methods

### 2.1. Patients and Tissue Samples

We retrospectively retrieved MLPS patients’ information from the established database at the Istituto Nazionale Tumori IRCCS “Fondazione G. Pascale”. Fifty eligible formalin-fixed paraffin-embedded (FFPE) specimens collected between the years 2001 and 2020 were taken into account. All patients had provided written informed consent for the use of tissue samples according to the institutional regulations. The study was approved by the Ethics Committee of the Istituto Nazionale Tumori IRCCS ‘Fondazione G. Pascale”. Histopathological diagnoses were reviewed according to the 2020 WHO classification criteria [2], based on clinical information, morphological criteria, and *DDIT3* break-apart FISH. Medical records were reviewed, and up-to-date information was collected to assess PFS.

### 2.2. Immunohistochemistry

Immunohistochemistry (IHC) was performed on 4 µm thin FFPE tissue sections as previously described [29], using automated slide stainers BenchMark (Ventana Medical System-Roche) and Leica Bond-III, (Leica Biosystems,), according to manufacturer’s instructions. To characterize lymphocytic infiltrate, CD3 (clone 2GV6, ready to use), CD4 (clone SP35, ready to use), and CD8 (clone SP57, ready to use) monoclonal antibodies (Roche) were applied on tissue sections for 15 min at 25 °C. Monoclonal antibody directed to nuclear transcription factor forkhead box P3 (FOXP3) (clone D2W8E, 1:250, Cell Signaling Technology) was utilized to identify regulatory T cells. CD68 (clone KP-1, ready to use, Roche), and CD163 (clone MRQ-26, Roche) monoclonal antibodies were employed to identify macrophages and M-2 like macrophages, respectively [33]. Intra-tumoral vascularization was assessed by using CD31 monoclonal antibody (clone JC70A, ready to use, Dako). Sections were counterstained with hematoxylin. 

### 2.3. Image Analysis

Slides were recorded by a light microscope connected to a video camera and analyzed by using the Axiovision 4.4 software (Carl Zeiss, Oberkochen, Germany). Quantitative analysis of immune infiltrates was conducted by two independent molecular pathologists (M.V.C., S.S.), blinded to clinical information. Sections were scored based on the average counts of positive cells or microvessels counted in the tumor areas, in five randomly selected fields/sample, each field having area of 0.785 mm^2^, at 200-x magnification.

### 2.4. Primary MLPS Cell Culture

A representative sample (∼1 cm × 1 cm) from the tumor excision of the high grade #37, #47, and low grade #48 and #50 MLPS patients were immediately minced with a scalpel under sterile conditions, incubated with 1 mg/mL collagenase XI (Sigma-Aldrich, #9407 Saint Louis, MO, USA) for 3 h at 37 °C under gentle agitation, and subsequently subject to filtration with sterile nylon filters (40 µm mesh) as described [34]. After recovering, cells were cultured in 6-well multi-dish plates in Dulbecco Modified Essential Medium (DMEM, Cytiva HyClone™ #SH30081.01, (Cytiva, Marlborough, MA, USA) with the addition of 10% fetal bovine serum (FBS, Cytiva HyClone™ #SV30160.03), penicillin (100 U/mL, and streptomycin (100 μg/mL, Cytiva HyClone™ #SV30010, Cytiva, Marlborough, MA, USA). Isolated cell clusters obtained from only #37 and #47 high grade tumor tissues were further amplified in growth medium until an adherent, homogeneous, round population was obtained. Mesenchymal phenotype was identified by the lack of staining for cytokeratin AE1/AE3 (sc-81714) and by the positive staining for vimentin (Clone V9, sc-6260), all antibodies being purchased by Santa Cruz (Dallas, TX, USA). For in vitro experiments, the #37 and #47 primary MLPS cells were amplified for further eight and fifteen passages, respectively. 

### 2.5. Isolation of Blood Monocytes

Buffy coats were obtained from healthy blood donors at Transfusion Medicine of the National Cancer Institute of Naples, after informed written consent. Peripheral blood mononuclear cells (PBMC)s, and serum were individually collected. PBMCs were harvested by density gradient centrifugation as previously described [29], using the Lympholyte-poly Cell Separation Media (Cedarlane Laboratories, #CL5015, Cedarlane, Burlington, ON, Canada) according to the manufacturer’s instructions. Monocytes were isolated from PBMCs by positive selection of CD14+ cells using the Monocyte Isolation Kit II purchased by Miltenyi Biotec, #130-091-153 (79% pure by visual and cytofluorimetric analysis) and transferred to tissue culture plates in RPMI-1640 medium (Cytiva HyClone™ #SH30096.01, Cytiva, Marlborough, MA, USA), supplemented with 10% autologous human serum, penicillin (100 U/mL), and streptomycin (100 μg/mL). 

### 2.6. Non-Contact Co-Cultures and Collection of Conditioned Media

Non-Contact co-cultures were carried out as described [29], using 24-multiwell plates and transwell polyethylene terephthalate permeable supports that allow the exchange of soluble factors purchased by Corning (Falcon^®^, #353095, NY USA). Briefly, human monocytes (1 × 10^6^ cells/well) were seeded in the lower compartment in RPMI-1640 medium supplemented with 10% heat inactivated human serum. MLPS cells (2 × 10^5^ cells/well) suspended in growth medium were seeded on the filter top and incubated for 72 h, at 37 °C with 5% CO_2_, changing medium every other day. Then, MLPS cells were removed, and conditioned medium (CM) from monocytes was achieved by adding serum-free RPMI-1640 medium. After 18 h, CM was recovered, centrifuged twice at 2200 rpm for 5 min at 4 °C, and aliquots stored at −80 °C. 

### 2.7. Cytofluorimetric Analysis

Monocytes recovered from co-cultures with/without MLPS cells were analyzed by flow cytometry as described [29], using PE-conjugated anti-CD68 REAfinity™ (Miltenyi Biotec #130-114-460) and APC-conjugated anti-CD163 REAfinity™ (Miltenyi Biotec, #130-112–129, Bergisch Gladbach, Germany) antibodies. Samples were acquired with the BD FACSCanto II (BD Biosciences, Franklin Lakes, NJ, USA), and data analyzed by the FlowJo v10.0.7 software (Tree Star, Inc, Ashland, OR, USA), after gating on the myeloid population in the FSC/SSC plot. Values were expressed as the percentage of each specific marker over median fluorescence intensity of the unstained cells.

### 2.8. Dot Blot Array

The relative levels of CC-chemokine ligand 2 (CCL2), IL-10 and IL-12 in CMs secreted by monocytes after co-culturing with primary MLPS cells were analyzed using the dot blot Human Cytokine Array Kit (#ARY005, R&D Systems, Milan Italy), according to the manufacturer’s instructions. Briefly, 500 mL CM were applied on each membrane, and signals were detected using the streptavidin-horseradish peroxidase and chemiluminescent detection reagents as described [29].

### 2.9. Invasion Kinetic of MLPS Cells Monitored in Real Time

Cell invasion assay was assessed using the xCELLigence Real Time Cell Analysis (RTCA) technology (Acea Bioscience) and E-16-well plates (#05469830001) as described [35]. Bottom wells were coated with 20 μg/well matrigel (Corning^®^ #356231) diluted in serum-free medium. Matrigel was allowed to polymerize for 1 h at 37 °C prior to seed MLPS cells (1 × 10^4^ cells/well) suspended in CM from monocytes pre-cultured with MLPS cells or CM from monocytes alone, the last employed as a control. In both cases, CMs were supplemented with 10% heat-inactivated human serum. Cells that cross matrigel adhere to the bottom of plates causing impedance changes which are proportional to the number of invading cells. The impedance value of each well was automatically monitored in real-time for 18 h and expressed as a cell index value.

### 2.10. 3D Organotypic Co-Cultures

Organotypic co-cultures of MLPS with monocytes were carried out as previously described [29]. Briefly, MLPS spheroids were obtained by pipetting MLPS cell suspension (5 × 10^3^ cells in 40 µL growth medium/well) into a Perfecta 3D Hanging Drop Plate (SigmaAldrich #HDP1096, Saint Louis, USA). Collagen/fibroblast matrix was obtained by suspending human dermal fibroblasts (Lonza #CC-2511) (1 × 10^5^ cells/sample) in 250 μL alpha Minimum Essential Medium (Cytiva HyClone™ #SH30024.01, Cytiva, Marlborough, MA, USA), containing 250 μL heat-inactivated serum and 2 mg/mL Type I Collagen (Cell Application Inc., #124–25, San Diego, CA, USA), with/without monocytes (1 × 10^5^ cells/well). MLPS spheroids were embedded into the collagen/fibroblast mixture, and images were acquired with an inverted microscope at 50× magnification for 7-days.

### 2.11. MLPS Cell Proliferation 

This assay was performed using the xCELLigence RTCA technology as described [36]. MLPS cells (2 × 10^3^/well) were seeded in 16-well E-plates in serum-free medium, CM from human monocytes pre-cultured with MLPS cells or CM from monocytes alone, the last as control. In all cases, media were supplemented with 2,5% heat-inactivated human serum. Gold microelectrodes placed on the bottom of plates detect impedance changes, which are proportional to the number of adherent cells and are expressed as Cell Index. The impedance value of each well was automatically monitored for 96 h and expressed as a Cell Index value.

### 2.12. Trans-Endothelial Migration

Trans-endothelial migration assays were performed using the xCELLigence RTCA technology as described [35]. Human Umbilical Vein Endothelial Cells (HUVEC)s, purchased by Lonza (#CC-2519), were employed between the third and the seventh passage and grown in Eagle Basal Medium (#CC31-56) supplemented with 4% FBS, 0.1% gentamicin, 1 µg/mL hydrocortisone, 10 µg/mL epidermal growth factor, and 12 µg/mL bovine brain extract packaged together (EGM-2 Endothelial Cell Growth Medium-2 BulletKit (LONZA #CC-3162, Basel, Switzerland). HUVECs (2 × 10^4^ cells/well) suspended in growth medium, were plated on E-16-well plates and allowed to grow for ~25 h until they formed a confluent monolayer, prior to seeding primary MLPS cells (2 × 10^3^ cells/well) suspended in serum-free medium, CM from human monocytes pre-cultured with MLPS cells, or in CM from monocytes alone. Medium and CMs were supplemented with 10% serum. When HUVECs are challenged with crossing cells, there is a drop in electrical resistance which is monitored in real-time for 5 h as the cell index changes due to crossing of the endothelial monolayer. The experiments were performed twice in quadruplicate.

### 2.13. Statistical Analysis

Data are expressed as the means ± SD of the number of the indicated determinations. Statistically significant differences were defined as *p* < 0.05. Multiple comparisons were performed by one-way ANOVA post hoc Dunnett *t*-test. Pearson’s correlation test was used to analyze the occurrence of any correlation between patient age, max tumor size, average counts of CD3, CD4, CD8, CD68, and CD163 positive cells and CD31 positive microvessels in tumor tissues from MLPS patients by using the SPSS 20.0 software (SPSS Inc., IBM New York, NY, USA). Kaplan–Meier analysis was used as appropriate to evaluate the PFS.

### 2.14. Ethics Statement

All experimental protocols were performed in accordance with guidelines of the Istituto Nazionale Tumori “Fondazione G. Pascale” IRCCS (Quality System n. LRC 6019486/QMS/U/IT-2015 certificated in conformity with UNI EN ISO 9001:2008). The research work with primary cell lines and MLPS tissues has been approved by Institutional Ethical Committee of Istituto Nazionale Tumori “Fondazione G. Pascale”-IRCCS, Naples, Italy (protocol 258/18, December 2018). 

## 3. Results

### 3.1. Phenotypic Characterization of Immune Cells Infiltrating MLPS Tissues

**Figure 1:**
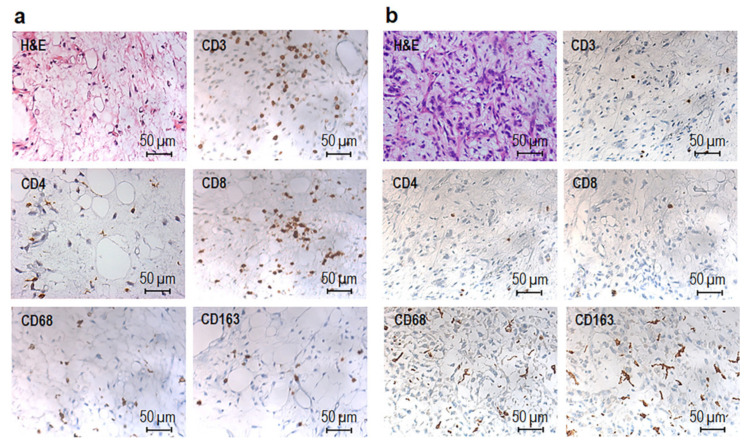
Immunohistochemical characterization of immune infiltrate in MLPS tissues. Representative images of H&E and IHC staining of CD3+, CD4+, CD8+, CD68+, and CD163+ positive cells in FFPE sections from a low grade MLPS (**a**) from patient #21 and high grade. MLPS tissues (**b**) from patient #4, acquired at 200× magnification.

To identify and quantify the cellular composition of the MLPS tissue immune infiltrates and tumor vascularization, fifty-patients collected between the years 2001 and 2020 were included in the study. At the time of diagnosis, the median age of patients was 51 years (range, 21–77 years), and their clinical-pathological characteristics are shown in Table 1 and Table S1. Patients had not received neo-adjuvant chemotherapy or radiotherapy before to be subjected to surgical resection. Histopathological diagnoses were reviewed according to the 2020 WHO classification criteria [2], based on clinical information, morphological criteria, and *DDIT3* break-apart FISH. Tumor tissues derived from the resection of primary tumors with the exception of one recurrence and two metastases and included twenty-six low grade and twenty-four high grade MLPS. Median tumor size was 13.8 cm, ranging between 2 and 35 cm (Table 1 and Table S1). Metastatic lesions occurred in two MLPS patients and six MLPS patients died of causes unrelated to the disease. PFS was calculated by reviewing the medical records of forty-three patients enrolled between 2001 and 2010, the others being enrolled between 2012 and 2020. IHC carried out on FFPE tissue sections allowed us to identify, quantify, and assess spatial distribution of tumor-infiltrating CD3+ T, CD4+ T helper and CD8+ T cytotoxic lymphocytes, FOXP3+ Treg lymphocytes, CD68+ macrophages, CD163+ alternatively activated M2-like macrophages, and CD31 positive microvessels. Both low and high grade MLPS tissues appeared infiltrated by T lymphocytes and macrophages, although to a different extent (Figure 1a,b). For each tissue sample, positive T cells, macrophages and microvessels were counted in five randomly selected fields/sample acquired at 200× magnification and the averages of lymphocytes macrophages and microvessels for each MLPS tissue (Table S2) were subjected to statistical analysis. High grade tumors were mainly infiltrated by CD68+ and CD163+ macrophages, whereas discrete amounts of CD3+ T, CD4+ T helper, and CD8+ T cytotoxic lymphocytes were observed in the perivascular areas of low grade MLPS (Figure 1a,b and Table S2).

**Figure 2:**
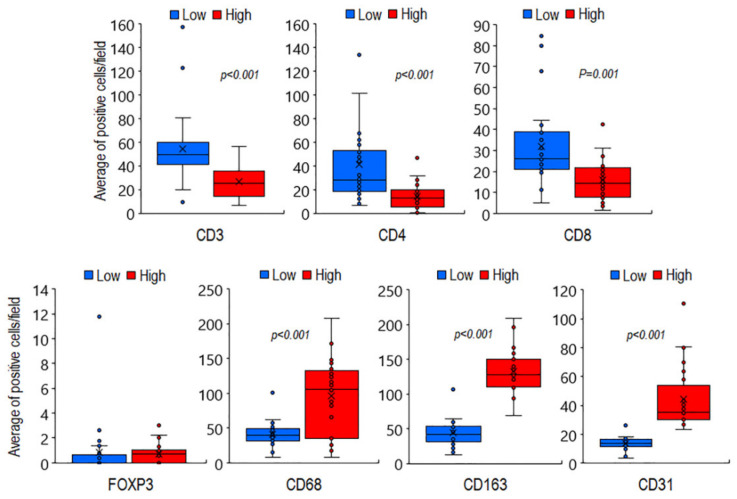
Correlation between immune cell infiltration and microvessel density in 26 low grade versus 24 high grade MLPS tissues. Box plots, showing variation in the average count of CD3+, CD4+, CD8+, FOXP3+, CD68+, and CD163+ cells as well as CD31 positive microvessels according to low or high histologic grade. Dark horizontal lines indicate the medians. Circles indicate outliers.

As shown in the Figure 2, statistically significant differences were found between the mean number of T lymphocytes counted in low grade MLPS tissues (n.54 CD3+, n.41 CD4+, and n.32 CD8+ cells/field), compared to high grade ones (n.24 CD3, n.14 CD4, and n.16 CD8 positive cells/field). Moreover, the differences assessed between the average counts of macrophages in high grade (n.91 CD68+ and n.140 CD163+ cells/field) versus low grade (n.41 CD68+ and n.44 CD163+ cells/field) MLPS tissues were statistically significant with *p* < 0.001 (Figure 2). 

**Figure 3:**
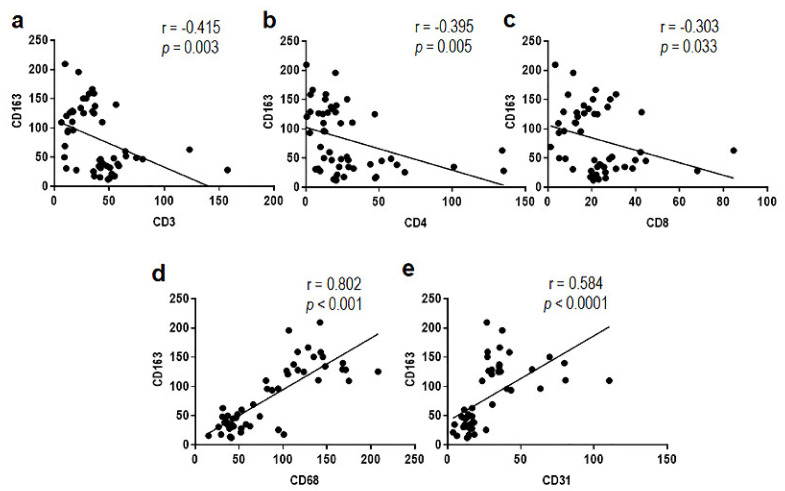
Pearson correlation between the average count of CD163+ macrophages and (**a**) CD3+ T lymphocytes, (**b**) CD4+ T helper lymphocytes, (**c**) CD8+ cytotoxic lymphocytes, (**d**) CD68+ macrophages, and (**e**) CD31+ microvessels in 50 MLPS tissue sections. Pearson correlation coefficients (r) are indicated.

Of note, few Treg lymphocytes were found to infiltrate both low and high grade MLPS tissues (Table S2), whereas a statistically significant increase of capillary network was found in high grade MLPS sections (median: 129 microvessels/field), compared with low grade tumors (median: 42 microvessels/field) (Figure 2 and Table S2). Since the number of infiltrating CD163 positive macrophages appeared to better discriminate high grade from low grade MLPS tissues, the occurrence of any relationship between CD163+ macrophages and patient age, maximal tumor size as well as average counts/field of CD3+, CD4+, CD8+, and CD68+ cells were subjected to Pearson correlation analysis (Table S3). We found that CD163+ macrophage counts positively correlated with CD68+ macrophages, microvessel densities (Figure 3) as well as with patient age, but negatively with CD3+, CD4+, and CD8+ lymphocyte counts (Figure 3 and Table S3).

Interestingly, patient ages were found to directly correlate with CD163+ macrophages but inversely with CD3+, CD4+, and CD8+ lymphocyte counts (Table S3). No correlation between tumor size and immune cell infiltration was found. (Table S3). Collectively, these observations seem to indicate that the aggressiveness of MLPS is modulated by its immune environment and that the alternatively activated M2 macrophages could support growth and invasive capability of MLPS cells. 

**Figure 4:**
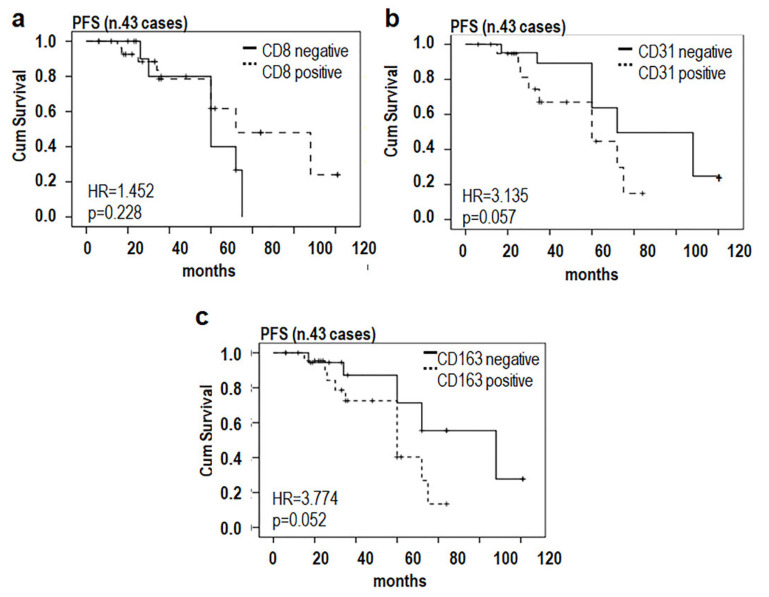
Prognostic implication of macrophages and microvessel density in MLPS. Kaplan–Meier analysis (Log-rank, Mantel–Cox test) used to evaluate the PFS, based on tumor-infiltrating CD8+ (**a**), CD31+ microvessels (**b**) and CD163+ cells (**c**) in 43 MLPS cases. Patients who were lost to clinical follow-up were censored from PFS at time lost to clinical follow-up (nine low and three high grade MLPS cases censored). Time was defined as the interval between the date of diagnosis and the date of disease recurrence or that of the last follow-up visit. HR, hazard ratio.

To further investigate on the relationship occurring between paucity of cytotoxic T-lymphocytes and abundance of alternatively activated M2 macrophages, the PFS of MLPS patients was subjected to Kaplan–Meier analysis (Log-rank, Mantel–Cox test). Average counts of CD8+ lymphocytes, CD163+ macrophages, and CD31+ microvessels were dichotomized as poorly infiltrated, negative (0–20 CD8+, 0–50 CD163+ average cell count/field or 0–25 CD31+ microvessels/field), or highly infiltrated, positive (>20 CD8+, >50 CD163+ average cell counts/field or >25 microvessels/field). PFS time was defined as the interval between the date of diagnosis and the date of first disease recurrence or, in the absence of any recurrence, that of last follow-up visit. Kaplan–Meier analysis revealed that patients whose tissues were mainly infiltrated by cytotoxic T lymphocytes displayed a trend toward a better PFS (HR = 1.452, *p* = 0.228) whereas a shorter PFS was associated to patients whose tissues were highly vascularized (HR = 3.135, *p* = 0.057) and/or massively infiltrated by CD163 positive macrophages (HR = 3.774, *p* = 0.052) (Figure 4), indicating that a relationship between the number of TAMs infiltrating tumor tissues and poor prognosis of MLPS patients does exist.

### 3.2. MLPS Cells Polarize Macrophages toward an M2-Like Pro-Tumor Phenotype

**Figure 5:**
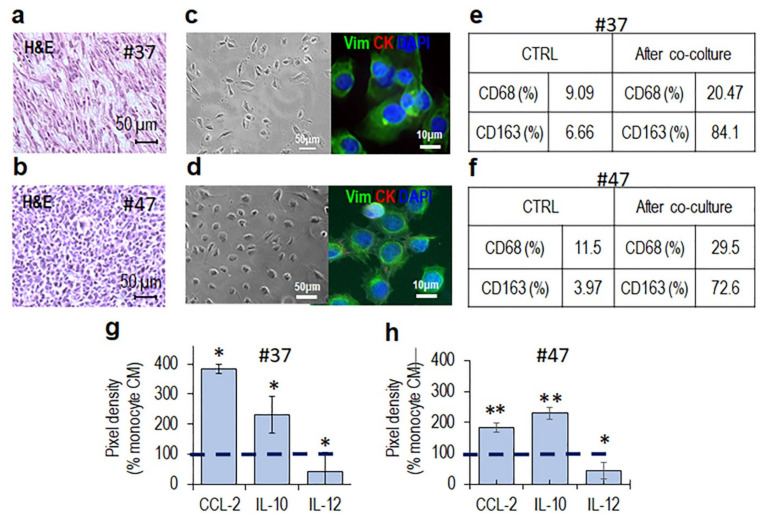
MLPS cells trigger M2-like polarization of monocytes in non-contact co-cultures. (**a**,**b**) Representative images of H&E staining of FFPE sections from #37 (**a**) and #47 (**b**) MLPS tissues acquired at 200× magnification. (**c**,**d**) Primary MLPS cells obtained by enzymatic digestion of 37 (**c**) and #47 (**d**) tumor tissues, visualized by phase contrast microscopy (left) and fluorescent microscopy after immunostaining with anti-vimentin and anti-cytokeratin Abs (right). Nuclei were stained blue with DAPI. Original magnifications: 200× (left) and 400× (right). (**e**,**f**) Human monocytes were co-cultured with #37 (**e**) and #47 (**f**) primary MLPS cells in an in vitro non-contact co-culture for 72 h and then analyzed for CD68 and CD163 expression by flow cytometry. (**e**,**f**) Percent variation of CD68 and CD163 on monocytes collected after non-contact co-culture, compared to control monocytes. (**g**,**h**) After co-cultures with #37 (**g**) and #47 (**h**) primary MLPS cells, CMs from monocytes were analyzed for the content of CC2, IL-10, and IL-12 by a dot plot assay. The pixel density of each spot was measured using NIH Image J 2.0 software developed by the US NIH, USA and positive control spots were used to normalize results between the membranes. The intensity of each spot was averaged over the duplicate spots and expressed as percentage of each cytokine or chemokine spontaneously secreted by control monocytes (monocyte CM), considered as 100% (dashed line). Data represent mean ± SD from three experiments performed in duplicate with * *p* < 0.05, ** *p* < 0.005.

Cancer cells have been recognized to polarize tissue infiltrating macrophages toward an M2-like pro-tumor phenotype that, in turn, orchestrates many steps of tumor progression by secreting proteases and growth and angiogenic factors, as well as cytokines and chemokines [29,37,38,39]. To investigate whether MLPS cells engage a crosstalk with monocyte/macrophages, co-cultures of primary MLPS cells and monocytes from healthy donors were set up. Representative MLPS high grade tumor samples from patients #37 (Figure 5a) and #47 (Figure 5b) were subjected to enzymatic digestion and the recovered cells amplified until two adherent and homogeneous cell populations were obtained (Figure 5c,d, left). The mesenchymal phenotype of both primary MLPS cells was assessed by the lack of staining for cytokeratin AE1/AE3 and by the positive staining for vimentin (Figure 5c,d, right). Primary #37 and #47 MLPS cells were co-cultured with human monocytes in an in vitro non-contact co-culture system. After 72 h co-culture, monocytes were recovered and analyzed by flow cytometry using CD68 and CD163 Abs that recognize macrophages and alternatively activated M2 macrophages, respectively, [29]. After co-culture with either #37 (Figure 5e) or #47 (Figure 5f) primary MLPS cells, monocytes express high levels of CD68 and significantly higher levels of CD163 as compared to control monocytes (Figure S1a,b and Figure 5e,f). Polarized M2-like-macrophages are documented to secrete higher levels of CCL2 and IL-10 and lower levels of IL-12, as compared to macrophages [40]. Accordingly, higher levels of the CCL2 and IL-10 as well as lower levels of IL-12 were found in the CM from monocytes pre-co-cultured with either #37 and #47 primary MLPS cells, compared to CM from control monocytes (Figure S1c,d and Figure 5g,h), supporting the notion that MLPS cells effectively promote M2-like polarization.

### 3.3. Monocytes Increase Spreading and Invasive Ability of MLPS Cells

**Figure 6:**
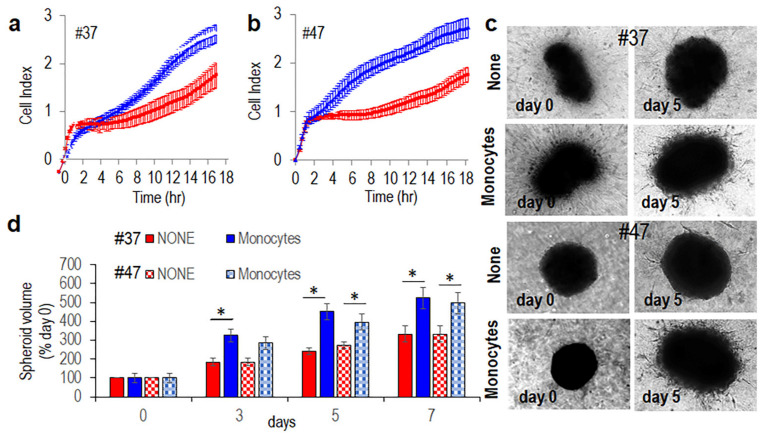
Monocytes pre-exposed to MPLS cells increases spreading of primary MLPS cells into matrices. (**a**,**b**). Primary #37 (**a**) and #47 (**b**) MLPS cells were suspended in CM from human monocytes pre-co-cultured with #37 and #47 (MLPS cells respectively (blue curves), or control monocytes (CTRL CM, red curves) and seeded onto matrigel-coated E-plates with serum added to a 10% final concentration. Matrigel invasion was monitored for 18 h by the RTCA xCELLigence technology. Data represent mean ± SD from a quadruplicate experiment representative of 2 replicates. (**c**,**d**). Spheroids containing primary #37 (**c**) or #47 (**d**) MLPS cells were embedded in the collagen/fibroblast mixture without (None), or with human monocytes. At the indicated times, images (**c**) were acquired at 50× magnification. (**d**) Time-dependent increase of spheroid size assessed by using the formula: V = D(d)^2^/2, where D and d are the major and the minor spheroid diameter, respectively. Data expressed as percentage of volumes assessed at time zero are the mean ± SD of two independent experiments, performed in duplicate. Statistical significance with * *p*  <  0.0001.

To understand whether monocytes/macrophages exert some effect on invasiveness of MLPS cells, we first determined the matrigel invasion rates of #37 and #47 primary MLPS cells exposed to CM from monocytes pre-cultured with MLPS cells or CM from control monocytes (CTRL CM), all supplemented with 10% heat-inactivated human serum, using the xCelligence technology. As shown in in Figure 6a,b CM from monocytes pre-cultured with #37 (Figure 6a) or #47 (Figure 6b) primary MLPS cells elicited a dramatic increase of MLPS cell invasion ability, as compared to CM from monocytes alone. Notably, the increase of MLPS cell invasiveness induced by soluble factors secreted by monocytes pre-exposed to MPLS cells is not due to cell proliferation increase, since cell index values recorded by proliferating MLPS cells exposed to diluents, CM from monocytes pre-cultured with MPLS cells or CM from control monocytes, all supplemented with 2.5% serum, generated overlapping curves (Figure S2). To further assess whether MLPS cells engage a crosstalk with monocyte/macrophages, organotypic 3D co-cultures that more accurately recapitulates key aspects of the architecture of solid cancers re-establishing morphological and functional features of the corresponding tissue in vivo, were set up. Primary #37 and #47 MLPS cells were allowed to form spheroids for 72 h. Once obtained, MLPS spheroids and monocytes were incorporated in a semi-solid matrix containing dermal fibroblasts. Then, spreading of MLPS cells into matrices and size of MLPS spheroids were monitored for 7 days. Fibroblast-dependent matrix deposition allowed spheroids to grow in control samples. Remarkably, the inclusion of monocytes cells into organoids caused a dramatic, time-dependent increase of #37 and #47 MLPS cell spreading and size of MLPS spheroids (Figure 6c). Measurement of spheroid volumes after 7 days, revealed that monocytes caused an about 35% increase of spheroid sizes (Figure 6d). 

### 3.4. Monocytes Increase Transendothelial Migration of MLPS Cells

**Figure 7:**
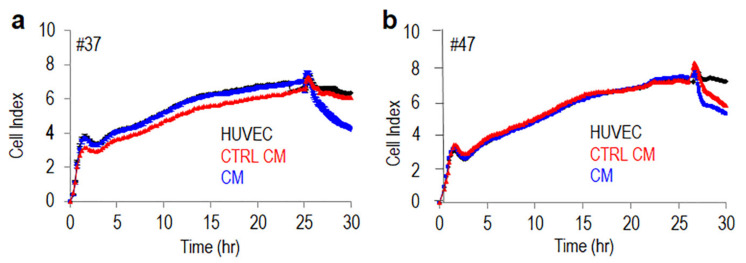
Monocytes pre-exposed to MPLS cells increases trans-endothelial migration of primary MLPS cells. HUVECs (1 × 10^4^ cells/well) suspended in growth medium were seeded onto E-plates and allow to grow for 25 h until they form a confluent monolayer. Then, primary #37 (**a**) and #47 (**b**) MLPS (2 × 10^3^ cells/well) suspended in CM from human monocytes pre-co-cultured with of MLPS cells, or control monocytes (CTRL CM) were seeded onto endothelial monolayers with serum added to a 10% final concentration. Cell index changes due to crossing of the endothelial monolayer were monitored in real-time for 5 h. The experiments were performed twice in quadruplicate.

Beside invading the extracellular matrix, tumor aggressiveness depends on tumor cell ability to entering the bloodstream. To analyze the ability of alternatively activated M2 macrophages to modify MLPS cell ability to cross endothelial monolayers, endothelial cells were allowed to grow in E-plates for about 25 h until they formed a monolayer, prior to seeding primary #37 or #47 MLPS cells suspended in complete medium, plus CM from monocytes pre-cultured with MLPS cells or CM from control monocytes, both supplemented with 10% serum. At this time, reduction of impedance values, due to invading cells that interrupt endothelial monolayers, was monitored for a further 5 h. According to invasion data, both #37 or #47 MLPS cells were able to disrupt the endothelial monolayer although to a different extent. In both cases, the presence of CM from monocytes pre-cultured with MLPS cells interrupted endothelial monolayers much more efficiently than in the presence of Control CM. Figure 7a,b).

Although the identification of soluble factors secreted by MLPS cells in the microenvironment milieu and responsible for M2-like polarization deserves further investigation, our findings indicate that MLPS cells really induce macrophage polarization in the direction of an M2-like pro-tumor phenotype and that once polarized, TAMs increase MLPS cell ability to spread and infiltrate surrounding tissues and ultimately entry of metastatic cells into the blood vessels.

## 4. Discussion

Although MLPS is relatively more sensitive to chemotherapy, compared with other soft tissue sarcomas, little advances have been made in the treatment of MLPS in the last three decades, and there is a strong request from patients of developing less toxic therapeutics that may improve patients’ outcome. It is largely documented that complex interactions between tumor cells and host immune responses in the TME may influence tumor evolution [21,22]. Thus, characterizing the MLPS immune microenvironment may provide prognostic and predictive biomarkers to enable the development of new therapeutic targets and strategies.

In this study, a quantitative evaluation of cellular components of the tumor immune infiltrate was conducted on fifty MLPS tissues in order to investigate the existence of any association of T-cell subtypes and tumor associated macrophages with the histologic grade and PFS. As the results presented here show, high grade MLPS tissues exhibited a CD3+, CD4+, and CD8+ T lymphocyte-poor phenotype whereas a discrete amount of CD3+, CD4+, and CD8+ lymphocytes was found to infiltrate low grade MLPS tissues. Dancsok and co-workers reported that single infiltration of CD8+ T cells in MLPS tissues correlates with better overall survival than simultaneous infiltration of CD8+ and FOXP3+ T cells [41]. Accordingly, we found that infiltration of CD8+ T cells associated to a better PFS, although very few T-reg cells were found to infiltrate both low and high grade MLPS tissues. It has been reported that trials with immune checkpoint inhibitors are delivered contradictory results since TME of MLPS is not much responsive to anti-PD-1 monotherapy [30]. In this regard, we may speculate that forcing T-cell recruitment into high grade MLPS tissues could ameliorate the response of MLPS patients to checkpoint inhibitors. For instance, immunotherapy targeting the cancer-testis antigen (NY-ESO-1) which is aberrantly expressed in MLPS [42] and has been reported to elicit both humoral and specific CD8+ cytotoxic T-cell immune responses [43,44] could improve the survival of patients affected by high grade MLPS. Furthermore, it has to be taken into account that most studies lump together different sarcoma subtypes that might exhibit immunological differences [45] and were carried out on different tissue areas, including tissue microarrays that may poorly represent the heterogeneity of histological pictures, compared to larger tumor sections.

In the TME, TAMs have been documented to promote tumor progression exerting immunosuppressive activities [38,46,47,48,49]. With regard to MLPS, the occurrence of a correlation between the infiltration of M2-like TAMs and poor prognosis was described for the first time by Nabeshima and co-workers on 78 MLPS samples [50]. Using immunohistochemistry for CD68 and CD163, they found that a high infiltration of either CD68^+^ macrophages and CD163^+^ M2-like TAMs associates with decreased overall survival and that conditioned medium from macrophages stimulates MLPS cell migration and invasion by activating the epidermal growth factor receptor (EGFR) [50]. We carried out a quantitative analysis of macrophages infiltrating 50 MLPS tissues by using CD68 as a macrophage marker and CD163 as a marker of alternatively activated M2 macrophages. According to Nabeshima and co-workers, we found that CD163+ alternatively activated M2 macrophages massively infiltrated high grade MLPS tissues, and their average counts positively correlated with patient age, but negatively with CD3+, CD4+, and CD8+ lymphocyte counts. Of note, patients whose tissues were heavily infiltrated by CD163+ macrophages showed a significant shorter PFS, compared with patients whose tissues were slightly infiltrated by macrophages, supporting the notion that TAMs negatively affect the prognosis of MLPS patients.

It is documented that TAMs originate from circulating monocytes that infiltrate tumor tissues differentiating into macrophages [51]. After monocyte recruitment in the solid tumors, a variety of signaling molecules, transcription factors, epigenetic mechanisms, and post-transcriptional regulators promote differentiation of the infiltrating monocytes, leading to “tumor-educated” macrophages which may exert immune-suppressive and pro-tumoral effects [52,53,54]. TAMs constitute a large portion of the tumor mass and regulate multiple aspects of cancer progression including matrix remodeling, tumor-associated angiogenesis, and immune surveillance [55,56]. 

Here, by non-contact co-cultures and 3D organotypic co-cultures, the last reproducing TME in a 3D-environment, we document the existence of a crosstalk occurring between primary MLPS cells and monocytes as MLPS cells trigger the differentiation of monocytes toward an M2-like anti-inflammatory phenotype, and M2-like macrophages increase spreading and invasiveness of MLPS cells into the matrices. Considering that TAM infiltration sustains tumor progression, many efforts have been made to prevent the recruitment of monocytes into tumor tissues, or to counteract their M2-like polarization, or, alternatively, to force their phenotype toward a M1 pro-inflammatory phenotype [57,58]. This is the case of the CCL2/CCR2 axis, which plays a role in the recruitment of monocytes in tumors: the CCR2 antagonist (PF-04136309) enhanced anti-tumor immunity, decreased tumor growth, and reduced metastasis in an orthotropic model of pancreatic cancer [59]. Trabectedin, approved for the treatment of MLPS, has been reported to reduce TAM density and decrease angiogenesis in mouse tumor models and in MLPS specimens [20]. Also, *all-trans* retinoic acid has been reported to reduce the number of pulmonary metastatic nodes of osteosarcoma cells in mice by inhibiting the M2 polarization of TAMs [60]. In the past years, we developed the retro-inverso peptide Ac-(D)-Tyr-(D)-Arg-Aib-(D)-Arg-NH2, named RI-3 that behaves, in vitro and in vivo, as a strong inhibitor of cell migration [35,61]. We have recently shown that following subcutaneous injection of primary chondrosarcoma cells in nude mice, a daily treatment with 6 mg/Kg RI-3 significantly reduced recruitment and infiltration of monocytes into tumors as compared to ones from untreated animals [29]. We are planning to investigate whether RI-3 exert similar effects on the monocyte recruitment into MLPS tissues in murine models. 

Many pieces are still missing to clarify the complicated puzzle of the neoplastic strategy of sarcomas. This study has been developed retrospectively and over a period of approximately 20 years. We hope that our findings could help to understand the interplay between microenvironment, immune response, and MLPS cells. In this regard, we foresee that the identification of soluble factors secreted by MLPS cells in the microenvironment milieu, and responsible for M2-like polarization, could lay down the bases for development of new-targeted therapies aimed to counteract TAM pro-tumor functions.

## 5. Conclusions

This study was aimed to quantify and functionally characterize the cellular composition of the tumor immune infiltrate in a large cohort of MLPS tissues and to explore the contribution of macrophages in promoting invasiveness of MLPS cells. Our data show that high grade MLPS tissues exhibit T lymphocyte-poor and M2-like macrophage-rich phenotypes and that a high M2-like macrophage infiltration associates to a shorter PFS. We also found that, when co-cultured with MLPS cells, macrophages exhibit predominantly a M2-like pro-tumor phenotype which sustains invasion capability of MLPS cells. Although soft tissue sarcomas have long been considered “immune cold” tumors, recent reports have shown a high degree of heterogeneity of the immunogenic features of these tumors, including MLPS. We are confident that our findings could help to understand the interplay between microenvironment, immune response, and MLPS cells. In this regard, the identification of soluble factors secreted by MLPS cells in the microenvironment milieu, and responsible for M2-like polarization, could allow the development of new-targeted therapies aimed to ameliorate the survival of patients affected by high grade MLPS.

## Figures and Tables

**Table 1:** Clinical and histologic findings of enrolled MLPS patients.

Myxoid Liposarcomas	Primary	47 (94%)
	Recurrence	1 (2%)
	Metastasis	2 (4%)
Age (years)	Mean	51.66
	<60	33 (66%)
	>60	17 (34%)
Sex	Male	27 (54%)
	Female	23 (46%)
Size (cm)	Mean	138.302
	<10 cm	14 (28%)
	>10 cm	30 (60%)
	Unknown	6 (12%)
Tumor location	Axilla	3 (6%)
	Chest wall	1 (2%)
	Abdomen	1 (2%)
	Pelvis	1 (2%)
	Gluteus	2 (4%)
	Thigh	29 (58%)
	Knee	1 (2%)
	Leg	12 (24%)
Histological grade	Low	26 (52%)
	High	24 (48%)
Follow up (ten years)	None	18 (36%)
	Recurrence	25 (50%)
	Unknown	7 (14%)

## Data Availability

All data generated during this study are available within the article and its supporting information. Further details are available from the corresponding author on reasonable request.

## Supplementary Materials

The following are available online at https://www.mdpi.com/article/10.3390/cancers13133298/s1. Table S1: Clinicopathological findings of enrolled MLPS patients. Table S2: Immunophenotypic characteristics of immune infiltrate in MLPS tissues. Table S3: Pearson correlation coefficients referred to the correlation between patients age; max tumor size; averages of CD3, CD4, CD8, CD68, and CD163 positive cells in tumor tissues from MLPS patients. Figure S1. Contribution of primary MLPS cells in promoting M2-like polarization of human monocytes in non-contact co-cultures. Figure S2. Conditioned media from human monocytes co-cultured with primary MPLS cells do not modify proliferation rate of MPLS cells.
