# Supplementary material for: Crosstalk between Macrophages and Myxoid Liposarcoma Cells Increases Spreading and Invasiveness of Tumor Cells
Source: Cancers (Basel). 2021 Jun 30;13(13):3298. doi: 10.3390/cancers13133298 (PMC8268435; doi:10.3390/cancers13133298)
Supplement: Supplementary file 1 [file cancers-13-03298-s001.zip › cancers-1277441-supplementary.pdf]

Table S1. Clinicopathological findings of enrolled MLPS patients.

Patient	Age (yr)	Gender	Site	Max ⌀ (cm)	P/R/Mt	Grade[1]	PFS (mo)
1	46	M	thigh dx	15	P	Low	120
2	67	F	axilla sx	ND	P	High	24
3	55	M	pelvis	20	P	High	6
4	36	M	thigh sx	18	P	High	36
5	42	F	thigh dx	16	P	High	33
6	34	M	thigh dx	3.5	P	High	62
7	51	F	thigh dx	17	P	High	22
8	49	M	thigh sx	16	P	Low	ND
9	75	M	thigh dx	ND	P	High	35
10	63	M	gluteus sx	8	P	Low	18
11	52	F	thigh sx	ND	P	Low	ND
12	63	M	thigh dx	18	P	High	12
13	36	M	axilla sx	11	P	Low	34
14	56	F	thigh sx	ND	P	Low	120
15	51	M	leg sx	8	P	High	20
16	51	F	leg dx	8	P	Low	27
17	23	F	thigh sx	19	P	Low	33
18	39	M	thigh dx	21	P	Low	36
19	55	F	thigh sx	12	P	Low	17
20	35	F	leg dx	7	P	Low	120
21	55	M	thigh dx	19	P	Low	120
22	77	F	leg sx	4	P	High	120
23	26	M	thigh sx	12	P	High	35
24	21	M	thigh sx	14	P	Low	120
25	69	F	thigh sx	4.5	P	High	75
26	44	M	leg sx	17	P	Low	120
27	71	F	leg sx	ND	P	High	15
28	25	F	leg dx	14	P	Low	120
29	39	M	leg sx	11	P	Low	120
30	48	F	thigh dx	5.5	P	High	120
31	61	M	thigh sx	9	Mt	Low	120
32	30	M	gluteus dx	15	P	Low	120
33	43	F	knee sx	2	R	Low	ND
34	72	M	leg sx	20	P	High	120
35	49	M	thigh sx	9.2	P	Low	120
36	63	F	thigh sx	11	P	High	120
37	38	F	leg sx	14	P	High	120
38	69	M	leg sx	20	P	Low	120
39	50	F	thigh sx	15	P	Low	120
40	61	F	thigh dx	35	P	Low	19
41	65	M	abdomen	14	Mt	High	ND
42	82	F	leg dx	20	P	High	23
43	49	F	axilla sx	ND	P	Low	6
44	59	M	thigh dx	22	P	High	30
45	77	M	chest wall	9.5	P	High	26
46	40	M	thigh sx	20	P	High	25
47	62	M	thigh sx	10	P	High	ND
48	43	M	thigh sx	7	P	Low	ND
49	51	F	thigh sx	18	P	High	48
50	65	F	thigh sx	15	P	Low	ND

[1] Grade was assessed according to the WHO 2020 classification. PFS, Progression Free Survival (mo: months) from the diagnosis up to 10 years; F, female; M, male; P, primary; R, recurrence; Mt, metastasis; ND, Not Determined.

Table 2. Immunophenotypic characteristics of immune infiltrate and microvessel density in MPLS tissues.

Patient	Grade	CD3	CD4	CD8	Foxp3	CD68	CD163	CD31
1	Low	54	24	28	0	31	48.5	9.6
2	High	9.6	10	1.33	0	66	69.2	30.6
3	High	14.4	8.8	16.6	0	104	126.67	28
4	High	17	15	13.2	0.6	116.8	128	35.2
5	High	36	13.6	31	0	116.4	159.33	27.4
6	High	29	28.2	28.4	3	134.8	150.67	27.2
7	High	37	17.2	27.25	0	112	137.6	35.4
8	Low	64.6	16.2	42.2	0	52.8	60.4	11.4
9	High	34.8	20.3	42.26	0	171.2	128.67	30.2
10	Low	58.6	101	35	0	57.75	35.4	15.4
11	Low	48.8	20.7	20.66	0	41.2	12.33	13.4
12	High	22	20.2	11.6	0	106.4	196	37.4
13	Low	54.8	26	19.4	0.4	100.8	18	18.25
14	Low	44.2	44	23.5	0	35.4	39.4	17.4
15	High	12.2	2.67	5	2.2	87.6	93.6	43.4
16	Low	42.2	8.4	31	2.6	43.6	32	12.2
17	Low	40.6	29.2	25.6	0	41.4	35.2	4.8
18	Low	41.6	47.3	26.33	0.6	15.2	16	6.4
19	Low	20	9.8	19.75	0	39	28.2	15.4
20	Low	47.8	22.8	22.6	0.6	36.4	35.33	12
21	Low	50.25	19	23.2	0	39.8	14	14
22	High	34.6	4.6	21.75	0.8	128.4	166.67	35.6
23	High	26.6	11.6	21.25	1	208	125.6	36.2
24	Low	42.2	17.6	21.2	0	46.4	46.8	11.2
25	High	31.5	3.2	9.2	0.8	142.8	158.67	42.2
26	Low	52.33	21.4	22	0	52	21.8	3.8
27	High	36	47	22.6	0	123.6	125	34.8
28	Low	57.6	62.2	24	1.8	33.6	38.66	18
29	Low	157.6	135	68	11.8	52.4	28.5	16.6
30	High	24	18.4	18.6	1.2	147.8	134.4	35.2
31	Low	41.2	51.8	44.6	0	42.8	45.6	12.4
32	Low	123	134	84.6	1.4	31.4	63.333	16.8
33	Low	80.4	29	39.8	0	34.8	46.8	12.8
34	High	12.6	13	15	0	82	96	40.6
35	Low	65.2	28	29.2	0	48	52.25	14.2
36	High	11	0.6	13.6	0.6	105.4	121	30.2
37	High	6.4	23.8	4.8	0	174.8	109.6	23.6
38	Low	50.6	32.5	38.6	1.4	62.4	32.33	13.2
39	Low	10.8	6.6	11.4	0	26.6	31.2	10.8
40	Low	36.4	48	20.4	0	29.4	18	14.8
41	High	16.4	3	7.2	2	167.6	129.25	57.66
42	High	9.75	0.4	3.4	2.2	142	209.6	26.8
43	Low	9.2	12.4	5.2	0.2	37	50	15
44	High	16.4	31.8	12.4	1.3	140	110.8	80.5
45	High	43.6	12	12.75	1	80.6	110	110.4
46	High	26.4	13.2	20.6	0.8	145.2	150.8	69.6
47	High	17.2	12.2	7.4	0	94.4	96.4	63.4
48	Low	74.6	58.2	80	0.5	73.6	49	16.8
49	High	56.2	20.8	16.4	0.8	168	140	79.75
50	Low	35.6	67.5	26.2	0	94.66	26	26.33

Average of CD3, CD4, CD8, FoxP3, CD68 CD163 positive cells CD31 positive microvessels for field, counted in 5 field/sample at 200 x magnification.

Table S3. Pearson correlation coefficients referred to the correlation between patients age, max tumor size, averages of CD3, CD4, CD8, CD68 and CD163 positive cells, and microvessel density in tumor tissues from MLPS patients.

		age	size	CD3	CD4	CD8	CD68	CD163	CD31
	Pearson Corr.		-.057	-.328*	-.239	-.254	.205	.319*	,362**
Age	Sig. (2-tailed)		.697	.020	.094	.075	.153	.024	,010
	n.		50	50	50	50	50	50	50
	Pearson Corr.	-.057		-.029	-.120	.055	.055	.142	,356*
Size	Sig. (2-tailed)	.697		.851	.436	.722	.722	.358	,018
	n.	44		44	44	44	44	44	44
	Pearson Corr.	-.328*	-.029		.775**	.802**	-.391**	-.415**	-,281*
CD3	Sig. (2-tailed)	.020	.851		.000	.000	.005	.003	,048
	n.	50	44		50	50	50	50	50
	Pearson Corr.	-.239	-.120	.775**		.721**	-.334*	-.395**	-,265
CD4	Sig. (2-tailed)	.094	.436	.000		.000	.018	.005	,063
	n.	50	44	50		50	50	50	50
	Pearson Corr.	-.254	.055	.802**	.721**		-.316*	-.303*	-,350*
CD8	Sig. (2-tailed)	.075	.722	.000	.000		.025	.033	,013
	n.	50	44	50	50		50	50	50
	Pearson Corr.	.205	.055	-.391**	-.334*	-.316*		.802**	,588**
CD68	Sig. (2-tailed)	.153	.722	.005	.018	.025		.000	,000
	n.	50	44	50	50	50		50	50
	Pearson Corr.	.319*	.142	-.415**	-.395**	-.303*	.802**		,584**
CD163	Sig. (2-tailed)	.024	.358	.003	.005	.033	.000		,000
	n.	50	44	50	50	50	50		50
CD31	Pearson Corr.	,362**	,356*	-,281*	-,265	-,350*	,588**	,584**	
	Sig. (2-tailed)	,010	,018	,048	,063	,013	,000	,000	
	n.	50	44	50	50	50	50	50	

Averages of CD3, CD4, CD8, CD68, and CD163 positive cells as well as CD31 positive microvessels for field. counted in 5 field/sample at 200 x magnification. assessed by using the SPSS 20.0 software. Green boxes: positive correlation; orange boxes: inverse correlation; n. the number of analyzed cases. * Correlation is significant at the 0.05 level (2-tailed). ** Correlation is significant at the 0.01 level (2-tailed).

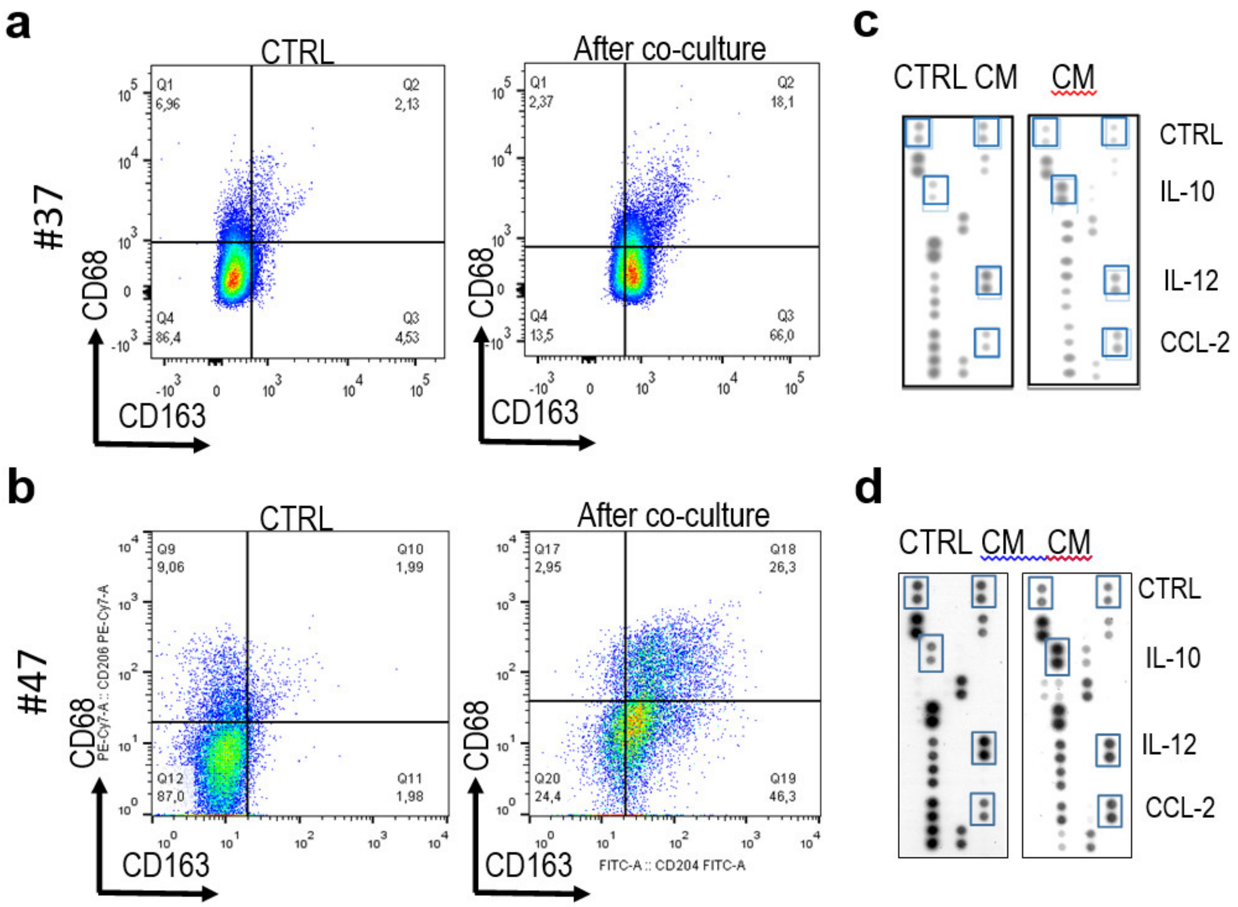

Figure S1. Primary MLPS cells promote M2-like polarization of human monocytes in non-contact co-cultures. **a–b**. Flow cytometry analysis of CD68 and CD163 on monocytes collected after non-contact co-cultures with #37 (a) and #47 primary MLPS cells, compared to control monocytes (CTRL). **c–d.** After co-cultures, primary MLPS cells were removed, and CM from monocytes pre-cultured with #37 (c) and #47 (d) primary MLPS cells (CM) was analyzed for the content of CC2, IL-10 and IL-12 by a dot plot assay using Cm from monocyes alone as control (CTRL CM).

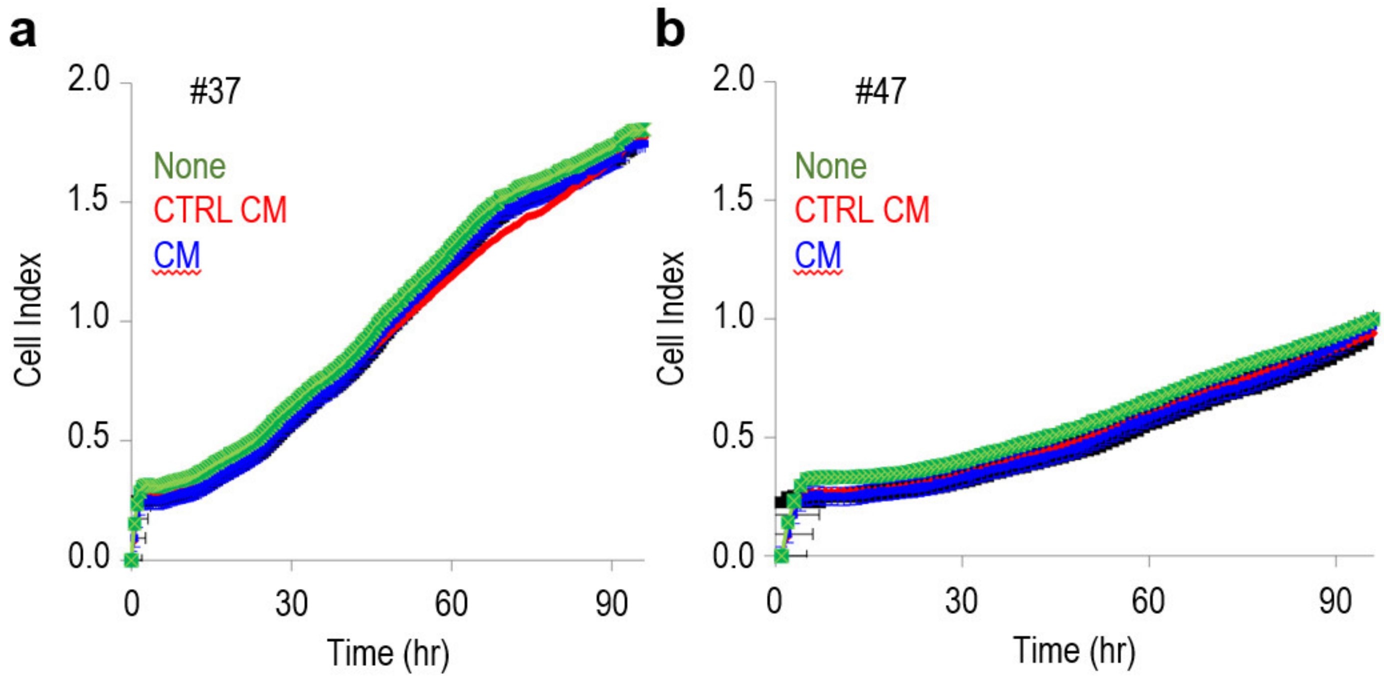

Figure S2. Conditioned media from human monocytes co-cultured with primary MPLS cells do not modify proliferation rate of MPLS cells. Primary MLPS cells derived from the tumor tissues #37 (a) and #47 (b) were suspended in diluents (None), CM from human monocytes pre-co-cultured with MLPS cells (CM) or CM from control monocytes (CTRL CM), all supplemented with 2,5% heat inactivated human serum, and seeded onto E-plates. Cell proliferation was monitored for 96 h with the RTCA xCELLigence technology. Data represent mean ± SD from a triplicate experiment representative of 3 replicates.
